# Association of Polygenic Score and the involvement of Cholinergic and Glutamatergic Pathways with Lithium Treatment Response in Patients with Bipolar Disorder

**DOI:** 10.21203/rs.3.rs-2580252/v1

**Published:** 2023-02-14

**Authors:** Azmeraw Amare, Anbupalam Thalamuthu, Klaus Oliver Schubert, Janice Fullerton, Muktar Ahmed, Simon Hartmann, Sergi Papiol, Urs Heilbronner, Franziska Degenhardt, Fasil Tekola-Ayele, Liping Hou, Yi-Hsiang Hsu, Tatyana Shekhtman, Mazda Adli, Nirmala Akula, Kazufumi Akiyama, Raffaella Ardau, Bárbara Arias, Jean-Michel Aubry, Lena Backlund, Abesh Kumar Bhattacharjee, Frank Bellivier, Antoni Benabarre, Susanne Bengesser, Joanna Biernacka, Armin Birner, Cynthia Marie-Claire, Pablo Cervantes, Hsi-Chung Chen, Caterina Chillotti, Sven Cichon, Cristiana Cruceanu, Piotr Czerski, Nina Dalkner, Maria Del Zompo, J. Raymond DePaulo, Bruno Etain, Stéphane Jamain, Peter Falkai, Andreas J. Forstner, Louise Frisén, Mark Frye, Sébastien Gard, Julie Garnham, Fernando Goes, Maria Grigoroiu-Serbanescu, Andreas Fallgatter, Sophia Stegmaier, Thomas Ethofer, Silvia Biere, Kristiyana Petrova, Ceylan Schuster, Kristina Adorjan, Monika Budde, Maria Heilbronner, Janos Kalman, Mojtaba Oraki Kohshour, Daniela Reich-Erkelenz, Sabrina Schaupp, Eva Schulte, Fanny Senner, Thomas Vogl, Ion-George Anghelescu, Volker Arolt, Udo Dannlowski, Detlef E. Dietrich, Christian Figge, Markus Jäger, Fabian Lang, Georg Juckel, Carsten Spitzer, Jens Reimer, Max Schmauß, Andrea Schmitt, Carsten Konrad, Martin von Hagen, Jens Wiltfang, Jörg Zimmermann, Till Andlauer, Andre Fischer, Felix Bermpohl, Vivien Kraft, Silke Matura, Anna Gryaznova, Irina Falkenberg, Cüneyt Yildiz, Tilo Kircher, Julia Schmidt, Marius Koch, Katrin Gade, Sarah Trost, Ida Haußleiter, Martin Lambert, Anja C. Rohenkohl, Vivien Kraft, Paul Grof, Ryota Hashimoto, Joanna Hauser, Stefan Herms, Per Hoffmann, Esther Jiménez, Jean-Pierre Kahn, Layla Kassem, Po-Hsiu kuo, Tadafumi Kato, John Kelsoe, Sarah Kittel-Schneider, Ewa Ferensztajn-Rochowiak, Barbara König, Ichiro Kusumi, Gonzalo Laje, Mikael Landén, Catharina Lavebratt, Marion Leboyer, Susan G. Leckband, Alfonso Tortorella, Mirko Manchia, Lina Martinsson, Michael McCarthy, Susan L. McElroy, Francesc Colom, Marina Mitjans, Francis Mondimore, Palmiero Monteleone, Caroline Nievergelt, Markus Nöthen, Tomas Novak, Claire O’Donovan, Norio Ozaki, Andrea Pfennig, Claudia Pisanu, James Potash, Andreas Reif, Eva Reininghaus, Guy Rouleau, Janusz K. Rybakowski, Martin Schalling, Peter Schofield, Barbara W. Schweizer, Giovanni Severino, Paul D Shilling, Kazutaka Shimoda, Christian Simhandl, Claire Slaney, Alessio Squassina, Thomas Stamm, Pavla Stopkova, Mario Maj, Gustavo Turecki, Eduard Vieta, Julia Veeh, Stephanie Witt, Adam Wright, Peter Zandi, Philip Mitchell, Michael Bauer, Martin Alda, Marcella Rietschel, Francis McMahon, Thomas G. Schulze, Vincent Millischer, Scott Clark, Bernhard Baune

**Affiliations:** University of Adelaide, AUSTRALIA; University of New South Wales; University of Adelaide; Neuroscience Research Australia; University Hospital LMU; University Hospital LMU; University Hospital LMU; University Hospital, LMU Munich; University of Bonn; National Institute of Mental Health; National Institute of Mental Health; University of California San Diego; University of California San Diego; Charité - Universitätsmedizin Berlin, Campus Charité Mitte; Dokkyo Medical University School of Medicine; Dokkyo Medical University School of Medicine; Hospital University Agency of Cagliari; Facultat de Biologia and Institut de Biomedicina (IBUB), Universitat de Barcelona, CIBERSAM; Geneva University Hospitals; University of California San Diego; University of California San Diego; Pôle de Psychiatrie, AP-HP, Groupe Hospitalier Lariboisière-F. Widal; Hospital Clinic of Barcelona; Medical University of Graz; Mayo Clinic; Medical University of Graz; INSERM UMR-S 1144; McGill University Health Centre; National Taiwan University Hospital; Research Center Juelich; Research Center Juelich; Max Plank Institute for Psychiatry; Poznan University of Medical Sciences; Medical University of Graz; University of Cagliari; Johns Hopkins University; Inserm U955, Psychiatry Genetics; Univ Paris Est Creteil, INSERM, IMRB; University Hospital LMU; University of Bonn, School of Medicine & University Hospital Bonn; Karolinska Institutet and Center for Molecular Medicine, Karolinska University Hospital; Mayo Clinic; Hôpital Charles Perrens; Alexandru Obregia Clinical Psychiatric Hospital; University of Tuebingen; University of Tuebingen; University Hospital Tübingen; University Hospital Tübingen; University Hospital Tübingen; University Hospital Tübingen; Ludwig Maximilian University of Munich; Ludwig Maximilian University of Munich; University Hospital Tübingen; Ludwig Maximillians Universitat Muenchen; Ludwig Maximillians Universitat Muenchen; Klinikum der Universität München; University Hospital Tübingen; University Hospital, LMU Munich; Klinikum der Universität München; University Hospital Tübingen; Ruhr University Bochum; Ruhr University Bochum; Ruhr University Bochum; Ruhr University Bochum; Ruhr University Bochum; Ruhr University Bochum; Ruhr University Bochum; Ruhr University Bochum; Ludwig-Maximilians-University (LMU) Munich; Ludwig-Maximilians-University (LMU) Munich; Ludwig-Maximilians-University (LMU) Munich; Ludwig-Maximilians-University (LMU) Munich; Universitätsmedizin Göttingen; Universitätsmedizin Göttingen; Universitätsmedizin Göttingen; Technical University of Munich, Klinikum rechts der Isar; Technical University of Munich, Klinikum rechts der Isar; University of Goettingen, Germany; Berlin School of Mind & Brain; Goethe University Frankfurt/M.; Goethe University Frankfurt/M.; University Medical Center Hamburg-Eppendorf; University Medical Center Hamburg-Eppendorf; University Medical Center Hamburg-Eppendorf; University Medical Center Hamburg-Eppendorf; University Medical Center Hamburg-Eppendorf; University Medical Center Hamburg-Eppendorf; University Medical Center Hamburg-Eppendorf; University Medical Center Hamburg-Eppendorf; University Medical Center Hamburg-Eppendorf; University Medical Center Hamburg-Eppendorf; University Medical Center Hamburg-Eppendorf; University Medical Center Hamburg-Eppendorf; McGill University; Institute of Human Genetics; Institute of Human Genetics; Institute of Human Genetics; Institute of Human Genetics; College of Public Health, National Taiwan University, Taipei, Taiwan; College of Public Health, National Taiwan University, Taipei, Taiwan; College of Public Health, National Taiwan University, Taipei, Taiwan; College of Public Health, National Taiwan University, Taipei, Taiwan; University Hospital of Würzburg; University Hospital of Würzburg; University Hospital of Würzburg; Landesklinikum Neunkirchen; Landesklinikum Neunkirchen; Hokkaido University Graduate School of Medicine; Gothenburg University; Gothenburg University; Karolinska Institutet; VA San Diego Healthcare System; VA San Diego Healthcare System; University of Naples SUN, University of Perugia; VA San Diego Healthcare System; VA San Diego Healthcare System; VA San Diego Healthcare System; Lindner Center of Hope / University of Cincinnati; Institut Hospital del Mar d’Investigacions Mèdiques; Max Planck Institute of Experimental Medicine, Göttingen, Germany; Johns Hopkins University; University of Salerno; School of Medicine & University Hospital Bonn; School of Medicine & University Hospital Bonn; Dalhousie University; Dalhousie University; University of Salerno; University Hospital Carl Gustav Carus, TU Dresden; University of Cagliari; University Hospital Frankfurt, Germany; University Hospital Frankfurt, Germany; McGill University; McGill University; Poznan University of Medical Sciences; Karolinska Institutet; Neuroscience Research Australia; Johns Hopkins University; University of Cagliari; University of Salerno; Universita degli Studi Di Cagliari; Universita degli Studi Di Cagliari; Universita degli Studi Di Cagliari; Universita degli Studi Di Cagliari; Charité - Universitätsmedizin Berlin, Campus Charité Mitte; National Institute of Mental Health; University of Campania “Luigi Vanvitelli”, Naples; McGill University; Hospital Clinic of Barcelona; University Hospital Frankfurt; University Medical Centre Mannheim; University of New South Wales; Department of Psychiatry and Behavioral Sciences, Johns Hopkins School of Medicine; Dalhousie University; Dalhousie University; Dalhousie University; University of Mannheim; National Institute of Mental Health Intramural Research Program; National Institutes of Health; Ludwig-Maximilians-University Munich; Mayo Clinic; University of Adelaide; The University of Munster

## Abstract

Lithium is regarded as the first-line treatment for bipolar disorder (BD), a severe and disabling mental disorder that affects about 1% of the population worldwide. Nevertheless, lithium is not consistently effective, with only 30% of patients showing a favorable response to treatment. To provide personalized treatment options for bipolar patients, it is essential to identify prediction biomarkers such as polygenic scores. In this study, we developed a polygenic score for lithium treatment response (Li+PGS) in patients with BD. To gain further insights into lithium’s possible molecular mechanism of action, we performed a genome-wide gene-based analysis. Using polygenic score modeling, via methods incorporating Bayesian regression and continuous shrinkage priors, Li+PGS was developed in the International Consortium of Lithium Genetics cohort (ConLi+Gen: N=2,367) and replicated in the combined PsyCourse (N=89) and BipoLife (N=102) studies. The associations of Li+PGS and lithium treatment response — defined in a continuous ALDA scale and a categorical outcome (good response vs. poor response) were tested using regression models, each adjusted for the covariates: age, sex, and the rst four genetic principal components. Statistical significance was determined at P< 0.05. Li+PGS was positively associated with lithium treatment response in the ConLi+Gen cohort, in both the categorical (P=9.8×10–12, R2=1.9%) and continuous (P=6.4×10–9, R2=2.6%) outcomes. Compared to bipolar patients in the 1st decile of the risk distribution, individuals in the 10th decile had 3.47-fold (95%CI: 2.22–5.47) higher odds of responding favorably to lithium. The results were replicated in the independent cohorts for the categorical treatment outcome (P=3.9×10–4, R2=0.9%), but not for the continuous outcome (P=0.13). Gene-based analyses revealed 36 candidate genes that are enriched in biological pathways controlled by glutamate and acetylcholine. Li+PGS may be useful in the development of pharmacogenomic testing strategies by enabling a classification of bipolar patients according to their response to treatment. Keywords: Polygenic score, pharmacogenomics, lithium, bipolar disorder, psychiatry

## Introduction

Bipolar disorder (BD) is a severe and often disabling mental health disorder that affects more than 1% of the population worldwide and is characterized by recurrent episodes of depression and mania^1^. BD accounted for 9.3 million disability-adjusted life years (DALYs) in 2017, and imposes a significant social and economic burden on society and healthcare systems^2, 3^. BD is associated with a significant somatic and psychiatric comorbidity^1^ and an increased risk of suicide^4^.

Since the discovery of lithium’s mood-stabilizing property in 1949^5^, it has been widely used as a first-line therapy for patients with BD^6, 7^. Lithium is effective in treating acute episodes of illness and reduces the risk of future recurrences of mania and depression^8^. It has also been shown to reduce the risk of suicide^9^. Despite these merits, the efficacy of lithium is highly variable, with about 30% of treated patients showing a favorable response while more than 30% of them have no clinical response at all^8, 10^. Thus far, the causes and predictors of such heterogeneity in treatment response are insufficiently understood. Genetic factors are thought to contribute, at least in part, to the large interindividual differences in response to lithium^10–15^. So far, only a few genetic studies have identified specific single nucleotide polymorphisms (SNPs) and candidate genes associated with patients’ response to lithium or treatment-related side effects^10, 11, 13–16^. Each employing a genome-wide association study (GWAS) approach, the Taiwan Bipolar Consortium found SNPs in the introns of GADL1 associated with lithium treatment response^17^, whereas the International Consortium on Lithium Genetics (ConLi^+^Gen) identified a locus on chromosome 21^10^, and a follow-up analysis uncovered additional variants within the human leukocyte antigen (HLA) region^14, 16^. Gene expression analysis of ConLi^+^Gen data also showed overexpression of genes involved in mitochondrial functioning in lithium responder patients, highlighting the electron transport chain as a potential target of lithium^18^.

In our recent work, we applied a polygenic score (PGS) modeling approach and demonstrated associations between a poor response to lithium and a high genetic loading for schizophrenia (SCZ)^14^, major depression (MD)^13^, and a meta-PGS combining both SCZ and MD^15^. Machine-learning models that combined clinical variables with the PGS of SCZ and MD has further improved the prediction of lithium treatment response, explaining 13.7% of the variance^19^.

Based on these previous results, translation of PGS testing into clinical practice requires the consideration of three important learnings. First, the PGS of a single phenotype (e.g., SCZ or MD) explains only a small proportion (< 2%) of the variability to treatment response in patients with BD^13, 14^, providing insufficient power for clinical use. Second, a meta-PGS from multiple related phenotypes has better predictive power than a PGS from a single phenotype^15^, suggesting the need to explore additional biological markers, including additional PGSs, that can either independently or together with existing PGSs better predict lithium treatment response. Third, developing polygenic markers with *direct* pharmacogenomic implications is essential, for example, a PGS for lithium treatment response (Li^+^_PGS_), which is perhaps biologically more related to lithium’s pharmacological actions than PGSs built for other clinical phenotypes (i.e. SCZ or MD; that may indirectly influence treatment response or symptom severity, but do not index pharmacogenetic signatures *per se*).

Here, we developed a novel Li^+^_PGS_ for lithium treatment response and applied gene-based pathway analyses to identify molecular mechanisms impacted by genetic variation in response phenotypes. Findings may assist in optimizing and personalizing the selection of mood stabilizers in patients with BD, and may point to novel molecular targets for future drug development.

## Methods And Materials

### Study Samples:

**Figure 1:**
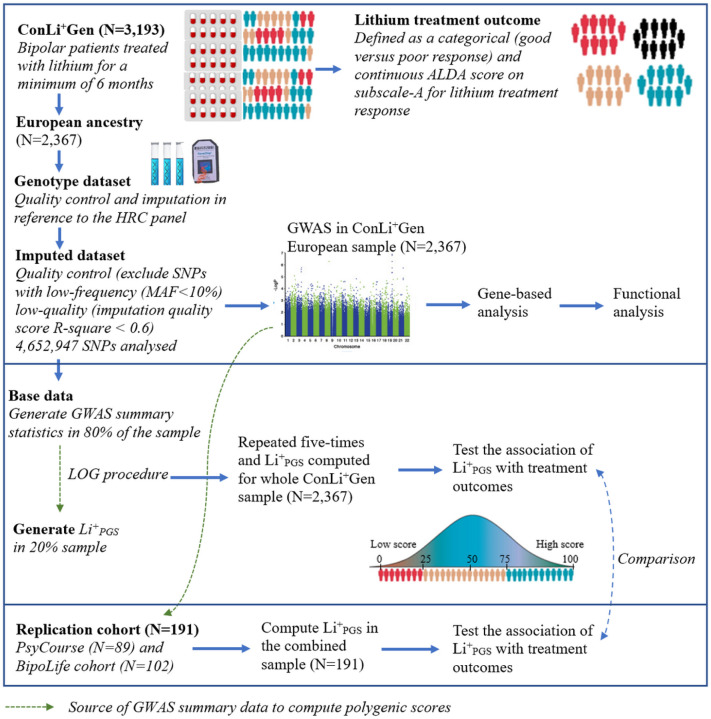
Overview of input datasets and steps of data analyses. ConLi^+^Gen = the International Consortium on Lithium Genetics, ALDA = Retrospective Criteria of Long-Term Treatment Response in Research Subjects with Bipolar Disorder scale, HRC= Haplotype Reference Consortium (HRC), SNPs = Single Nucleotide Polymorphisms, MAF=Minor Allele Frequency, GWAS = Genome Wide Association analysis, Li^+^_PGS_ = polygenic score for lithium treatment response, LOG=Leave-one-group out procedure; PsyCourse = Pathomechanisms and Signature in the Longitudinal Course of Psychosis study and BipoLife = German research consortium for the study of bipolar disorder.

For this study, we obtained genetic and clinical data from the International Consortium on Lithium Genetics (ConLi^+^Gen: N = 2,367), Pathomechanisms and Signature in the Longitudinal Course of Psychosis study (PsyCourse: N = 89), and BipoLife cohort (N = 102). Figure 1 shows the detailed steps of data analysis.

#### Discovery cohort

*ConLi*^+^*Gen* is a global collaboration of scientists established to study the pharmacogenomics of lithium treatment in patients with BD^10^. In the current study, we analyzed the genome-wide genotype and clinical data of 2,367 lithium-treated bipolar patients of European ancestry collected by 22 participating sites in 13 countries, including Australia (n = 122), Austria (n = 43), Czech Republic (n = 45), France (n = 210), Germany (n = 218), Italy (n = 255), Poland (n = 97), Romania (n = 152), Spain (n = 74), Sweden (n = 304), Switzerland (n = 57), Canada (n = 353) and the USA (n = 437)^10, 20^.

#### Replication cohort

To replicate Li^+^_PGS_ associations found in the discovery ConLi^+^Gen sample, we utilized datasets from PsyCourse and BipoLife where the study participants were of European ancestry. *PsyCourse* is a longitudinal multicenter study conducted from 2012 to 2019 in Germany and Austria, with up to four assessments at 6 monthly intervals. The study comprises 1,320 patients from psychotic-to-affective spectrum, of which, datasets from 89 patients with BD who received lithium treatment were obtained for this study^21^. *BipoLife* is a multicenter cohort study, established to investigate the biological basis of BD and patients’ response to treatment and being conducted across ten university hospitals in Germany (Berlin, Bochum, Dresden, Frankfurt, Göttingen, Hamburg, Heidelberg, Marburg, Munich and Tübingen) and the medical informatics section of the University of Göttingen^22^.

#### Target outcome

In both discovery and replication cohorts, patient’s treatment response was assessed using the “Retrospective Criteria of Long-Term Treatment Response in Research Subjects with Bipolar Disorder” scale, also called the ALDA scale^10^. The target outcome “lithium treatment response” was defined in categorical and continuous scales among patients who had received lithium for a minimum of 6 months^10^. The detailed procedures of ALDA scale measurement and its validity are described elsewhere^13, 14, 20^. Briefly, the ALDA scale measures symptom improvement over the course of treatment (A-score, range 0 – 10), which is then weighted against five criteria (B-score that assesses confounding factors, each scored 0, 1, or 2). Once we calculated the total score as ‘A-score minus B-score and setting negative scores to zero’, the categorical (good versus poor) lithium treatment response was defined at a cut-off score of 7, where patients with a total score of 7 or higher were considered as “responders”^10^. The continuous outcome for lithium treatment response was defined on subscale-A, but patients with a total B score greater than 4 or who had missing data on the totals of ALDA subscale-A or B were excluded^10^.

## Genotyping, Quality Control And Imputation

We obtained the genotype data assayed with different types of commercial SNP arrays across multiple cohorts^10, 21, 22^ and applied a series of quality control (QC) procedures before and after imputation using PLINK^23^. First, SNPs that had a poor genotyping rate (< 95%), strand ambiguity (A/T and C/G SNPs), a minor allele frequency (MAF) less than 1% or showed deviation from Hardy-Weinberg Equilibrium (P < 10^−6^) were removed. Then, individuals with low genotype rates (< 95%), who had sex inconsistencies (between the documented and genotype-derived sex), and who were genetically related were excluded.

### Imputation:

The genotype data passing QC were imputed on the Michigan server ^2424^ (https://imputationserver.sph.umich.edu) separately for each genotyping platform, using the Haplotype Reference Consortium (HRC) reference panel that consists of the largest available set (64,976 human haplotypes) of broadly European haplotypes at 39,235,157 SNPs^25^. For each cohort, imputation quality procedures were implemented to exclude SNPs of low-frequency (MAF < 10%) and low-quality (imputation quality score R-square < 0.6). From the imputed dosage score, genotype calls for the filtered SNPs were derived and common sets of 4,652,947 SNPs across the cohorts were merged using PLINK^23^.

## STATISTICAL ANALYSIS

We implemented polygenic score modeling, genome-wide SNP association, gene-based and functional analysis as described below.

### Genome-wide SNP association analysis:

Genome-wide SNP association analyses were performed on the binary lithium treatment response and continuous ALDA total score using logistic and linear regression models as implemented in PLINK software^23^, respectively. Each analysis was adjusted for the covariates: age, sex, chip type and the first four genetic principal components (PCs).

#### Polygenic score development

Using a polygenic score model constructed via Bayesian regression framework and continuous shrinkage (CS) prior on SNP effect sizes implemented in the PRS-CS software^26^, we built Li^+^_PGS_ for individuals of European descent who participated in the ConLi^+^Gen study and replicated the findings in the combined PsyCourse and BipoLife datasets. Polygenic scores were computed using PRS-CS to infer posterior SNP effect sizes under continuous shrinkage (CS) using GWAS summary statistics and an external linkage disequilibrium (LD) reference panel. For the current analysis, the precomputed LD pattern of the 1000 Genomes European reference panel^27^ and the discovery GWAS summary statistics were used to calculate PGS scores.

For the ConLi^+^Gen study, Li^+^_PGS_ was derived only for the European ancestry individuals (n = 2,367) using a five-fold leave-one-group out (LOG) procedure^28^ to remove discovery-target circularity. In each fold, 80% of the sample (n = 1,893) was used to generate GWAS summary statistics that were used as discovery for PGS calculation in the 20% left-out target sample (n = 474). The procedure was repeated five times by selecting a non-overlapping set of 20% left-out samples to calculate PGS for the entire cohort. Finally, Li^+^_PGS_ was computed for the PsyCourse and BipoLife participants using ConLi^+^Gen’s GWAS summary statistics (discovery sample) generated from the full European cohort (n = 2,367).

### Polygenic score association analysis:

To assess the association of Li^+^_PGS_ with lithium treatment response, a binary logistic regression model was applied for the binary outcome (good versus poor response to lithium treatment), and a Tobit analysis model (censored regression) was used for the continuous outcome (*ALDA total*)^29^. In addition, we divided the ConLiGen sample into deciles, ranging from the lowest polygenic load (1st decile, reference group) to the highest polygenic load (10th decile). Then, we compared BD patients in the higher polygenic load deciles (2nd – 10th deciles) with patients in the lowest polygenic load decile (1st decile). In both the binary and continuous outcomes, the proportion of phenotypic variance explained by Li^+^_PGS_ was computed as the difference in R^2^ of the model fit with Li^+^_PGS_ plus covariates, compared to the model t with only covariates. Each modeling analysis was adjusted for the covariates: age, sex, and the first four genetic PCs, and statistical significance was set at p < 0.05.

#### Gene-based and functional analysis

The gene-based analysis was based on summary statistics generated from the full European ancestry (n = 2,367) ConLi^+^Gen genome-wide SNP association analysis (see Fig. 3) and employed MAGMA (Multi-marker Analysis of GenoMic Annotation)^30^, a tool that uses a multiple regression approach to incorporate LD between markers and to detect multi-marker effects.

To explore the biological context of the genes discovered from the gene-based analysis, a pathway analysis was implemented using PANTHER (Protein ANalysis THrough Evolutionary Relationships; http://pantherdb.org/) classification system. PANTHER is designed to classify proteins (and their genes) into biological pathways^31^. To prepare the input genes for PANTHER, we selected genes that showed gene-level association with lithium treatment response (either with the categorical or continuous outcome) at MAGMA adjusted p-value < 0.001. This list of genes was entered into PANTHER version-17 which compares the proportion of input genes mapping to a biological pathway to the reference gene list from its databases. Molecular relationships previously experimentally observed in Homo sapiens (human) were included. The significance of the overrepresented PANTHER pathways was determined using Fisher’s exact test and later adjusted for multiple testing using the Bonferroni correction method. Significant associations were defined at p-value < 0.05.

## Results

### Sample Characteristics

The discovery analysis consisted of ConLi^+^Gen data obtained from 2,367 bipolar patients of European ancestry who had undergone lithium treatment for at least six months. The mean (sd) age of the patients was 47.5(13.9) years and 1,369 (57.8%) were female. In all, 660 (27.9%) of patients had a good response to lithium treatment (ALDA score ≥ 7). The mean (sd) ALDA score for ConLi^+^Gen participants was 4.1 (3.1). The replication analysis was based on a combination of the PsyCourse and BipoLife datasets (N = 191), whose mean (sd) age was 49.1(13.0) years. Of the 191 patients with BD, 48(25.1%) had a good response to lithium (Table 1).

### Associations Of Li With Lithium Treatment Response In Bipolar Patients

**Figure 2:**
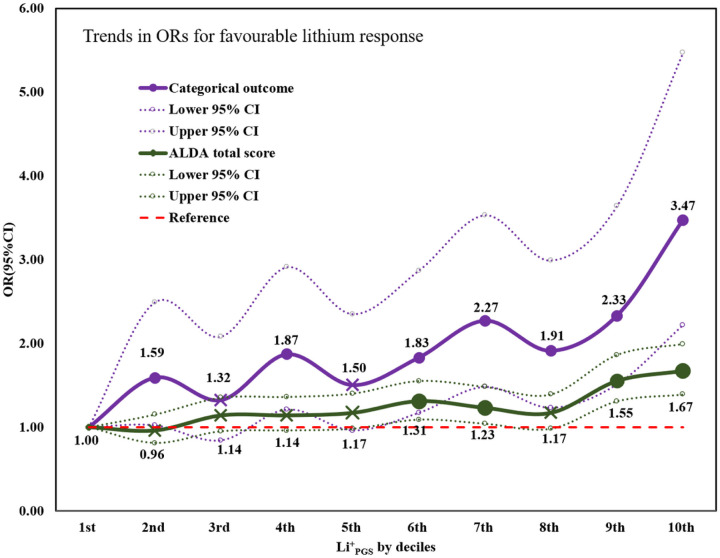
Trends in the odds ratios (ORs) for favourable treatment response to lithium for patients with bipolar disorder in the higher genetic loading for lithium responsive variants, deciles (2^nd^ to 10^th^) compared with patients in the lowest (decile 1^st^) of genetic loading for lithium response (n = 2,367). The X mark on the line plot indicates that the association is not statistically significant at that decile. *Abbreviations*: OR= odds ratio, CI=Confidence interval, Li^+^_PGS_ =polygenic score for lithium treatment response.

Using ConLi^+^Gen data, we found statistically significant associations between Li^+^_PGS_ and lithium treatment response — both in the categorical (P = 9.8×10^−12^, R^2^ = 1.9%) and continuous (P = 6.4×10^−9^, R^2^ = 2.6%) outcomes. Li^+^_PGS_ was positively associated with response to lithium treatment, with an adjusted odds ratio (OR) [95%CI]) of 1.39 [1.26, 1.54]. In other words, BD patients who carry a higher genetic loading for lithium responsive genetic variants, measured using the Li^+^_PGS_, have higher odds of favorable lithium treatment response, compared to patients carrying a low Li^+^_PGS_ load. Table 2 shows the association results of Li^+^_PGS_ and lithium treatment response in categorical and continuous outcomes. The odds of a favorable treatment response increased as the Li^+^_PGS_ increased, ranging from 1.59 fold [95%CI: 1.02–2.49] at the 2nd decile to 3.47 fold [95%CI: 2.22–5.47] at 10th decile, compared to the reference Li^+^_PGS_ at the 1st decile (Table 2). While there was an increasing trend in the odds of lithium treatment response across the deciles, the most significant prediction contrast was found at the ‘extremes’ (1st and 10th decile) which comprised of ~ 20% of the total cohort (Fig. 2). A replication PGS analysis in the combined PsyCourse and BipoLife samples found a statistically significant association of Li^+^_PGS_ with the categorical lithium treatment response (P = 3.9×10^−4^, R^2^ = 0.9%), but not with the continuous outcome (P = 0.13).

#### Genome-wide association, gene-based and functional analysis

**Figure 3:**
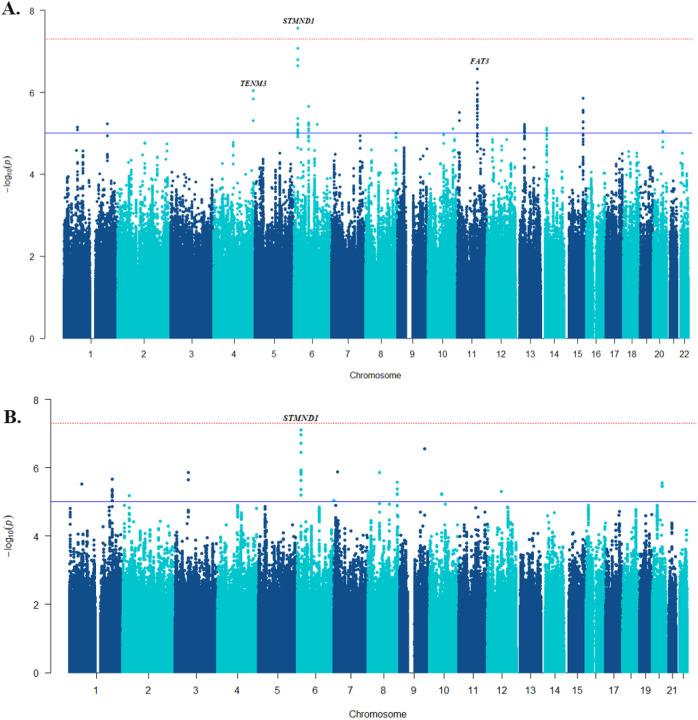
Manhattan plots showing the SNP-based GWAS results of lithium treatment response in patients with bipolar disorder; A) in the categorical outcome and B) continuous scale, highlighting the loci that showed genome-wide significance (orange). The −log10 (p-value) is plotted against the physical position of each SNP on each chromosome. The threshold for genome-wide significance (p-value < 5 × 10^–8^) is indicated by the red dotted horizontal line

After re-imputing the ConLi^+^Gen data in reference to the latest HRC genomes, we conducted GWASs on lithium response, both in categorical and continuous outcomes. This GWAS analysis identified a single locus with lead SNP rs9396756 located near the stathmin domain containing 1 (*STMND1*) gene that reached genome-wide significance for association with the categorical outcome (P = 2.7 ×10^−8^) and showed a suggestive association with the continuous ALDA score (P = 7.6 ×10^−8^) (Fig. 3). A follow-up gene-based analysis of the newly derived ConLi^+^Gen GWAS summary statistics found 36 candidate genes likely associated with lithium treatment response — assessed in either continuous or categorical outcomes (P < 0.001). In silico functional analysis of the 36 genes revealed enriched biological pathways including the muscarinic acetylcholine receptors 1 and 3 (P-value corrected for multiple testing = 0.026) and metabotropic glutamate receptor group III pathway (P = 0.043). These genes and pathways may have an impact on clinical response to lithium treatment and be potential molecular targets for lithium (Supplementary Fig. 1 and Supplementary Table 1).

## Discussion

This study presents findings from a comprehensive analysis of genetic and clinical data on lithium treatment response that involved the development of a polygenic score for lithium treatment response (Li^+^_PGS_), genome-wide SNP association and gene-based and functional analyses.

Since the publication of the first GWAS report by the ConLi^+^Gen team^10^, two landmark studies that independently showed the negative association of PGSs for SCZ and MD with lithium treatment response have been published^13–15^. The first study found that 10% of bipolar patients with the lowest polygenic load for SCZ were 3.46 times more responsive to lithium compared to 10% of patients with the highest genetic load for SCZ^14, 15^. Similarly, in the second study, 10% of patients who had the lowest genetic loading for MD were 1.54 times more responsive to lithium than 10% of patients with the highest genetic loading for MD^13, 15^. Nevertheless, each of these PGSs accounts for < 2% of the total variance to lithium treatment response^13^, suggesting the need to explore additional biological traits that can either independently, or in concert with existing PGSs better predict lithium response. Moreover, the previous PGSs for SCZ and MD are difficult to interpret in a pharmacogenomic context, making the development of a specific lithium response PGS necessary, which is assumed to be more likely to be associated with lithium treatment response and perhaps is biologically more related to lithium’s pharmacological actions.

In this novel study, we constructed a PGS for lithium response-Li^+^_PGS,_ a biological marker of direct pharmacogenomic relevance, and showed a positive relationship between a high genetic loading for lithium treatment response variants and long-term therapeutic response to lithium in patients with BD. We demonstrated that bipolar patients at the extreme tail end of the distribution have the strongest association, i.e. 10% of patients who carry high genetic loading for lithium responsive variants (10th decile) were 3.47 times more likely to respond to lithium compared to 10% of those with the lowest Li^+^_PGS_ (1st decile). These results indicated that Li^+^_PGS_ has the potential to help stratify bipolar patients according to predicted lithium response.

In a GWAS of lithium treatment response, we identified a locus near the *STMND1* gene, which encodes for proteins known to be involved in neuron projection development, and active in neuron junctions and cytoplasm. Previous analysis that employed the 1000 Genomes Project reference panel for imputation reported a suggestive association between genetic variants within the *STMND1* gene and lithium treatment response^10^.

Using our newly derived ConLi^+^Gen GWASs summary statistics as an input, we then carried out a gene-based analysis where several genetic variations were examined together for their association with lithium treatment response^30^. This approach found 36 potential target genes for lithium treatment that are enriched in the muscarinic acetylcholine receptors (mAChRs) 1 and 3 and the metabotropic glutamate receptor group III signaling pathways — well characterized biological pathways modulated by the most abundant neurotransmitters in the brain (glutamate and acetylcholine).

Acetylcholine is the central regulator of the mAChRs signaling pathways, which are subfamily of G protein-coupled receptor complexes located in the cell membranes of neurons and other cells that regulate fundamental functions of the central and peripheral nervous system including acting as the main end-receptor stimulated by acetylcholine released from postganglionic fibers in the parasympathetic nervous system^32^. The muscarinic antagonist scopolamine has antidepressant activity, while physostigmine, a cholinesterase inhibitor induces depressive symptoms, suggesting muscarinic receptors may play a role, not only in the pathogenesis of mood disorders, but also as therapeutic targets^33^. M1 and 3 receptors are localized in the cortex, hippocampus and substantia nigra and are known to activate protein kinase C (PKC), causing post-synaptic excitation. PKC is thought to be central in the molecular pathogenesis of BD.

Glutamate, the primary excitatory neurotransmitter in the central nervous system (CNS), exerts neuromodulatory actions via the activation of metabotropic glutamate (mGlu), a type of glutamate receptor that modulates synaptic transmission and neuronal excitability throughout the central nervous system^34^. Group III metabotropic glutamate receptors are largely presynaptically localized and downregulate neurotransmitter release from presynaptic terminals directly or indirectly. These receptors have a prominent expression in the brain, especially in the region of the hippocampus, and can lead to the inhibition of the cAMP cascade which is critical for the maintenance of long-term synaptic plasticity^35^. Growing evidence indicates that abnormalities in the glutamatergic system are implicated in the pathogenesis and treatment of mental health disorders^36^ including BD^37, 38^, SCZ^39^, neurodevelopmental disorders^40^, Huntington’s disease^41^ and Alzheimer’s disease^42^. Studies have reported SNPs of the mGluRs system associated with BD^43^, and in animal studies, lithium was found to alter intracellular calcium by modulating the activity of the metabotropic glutamatergic receptor system^44^. To summarise, findings from the genome-wide SNP association, gene-based and functional analysis highlight the possibility that mechanisms involving glutamate and acetylcholine signaling pathways might influence the therapeutic effects of lithium in patients with BD. Modulation of these pathways through genetic variants may disrupt or enhance lithium’s clinical effectiveness.

Our study has some limitations. First, while our findings were replicated in an independent small size sample, the fact that it was replicated in the binary outcome, but not in the continuous outcome indicates the need for a larger replication cohort. Second, because Li^+^_PGS_ was developed and evaluated in European-ancestry populations, the ndings should be replicated in a multi-ethnic population to gauge generalizability. Furthermore, the risks and bene ts of predictive models consisting of Li^+^_PGS_ should be evaluated in prospective studies. Third, Li^+^_PGS_ only explains about 2% of response variance in our cohort, and as such is comparable to PGSs from other phenotypes (SCZ, MDD) that have shown an association with treatment outcomes. On their own, these PGSs are not suited to clinical pharmacogenomic testing as they would not predict treatment response prospectively in individual patients. Prediction models combining Li^+^_PGS_ with other PGSs^13, 14^ and clinical characteristics^19, 45^ may improve the clinical utility of PGSs. Such models would then need to be tested in prospective studies and clinical trials. Forth, studies have shown that approaches to phenotyping of lithium treatment response can be improved using advanced methods such as machine learning ^46^. Employing a more precise phenotype definition may result in the identification of novel candidate genes implicated in lithium treatment response and ultimately the development of more informative Li^+^_PGS_.

In conclusion, we developed a unique lithium treatment response polygenic score (Li^+^_PGS_) that showed a positive association with better lithium treatment response in patients with BD. Our gene-based and functional analyses build upon the findings from existing molecular studies by linking lithium treatment response with muscarinic acetylcholine receptor signaling and metabotropic glutamate receptor pathways. Further pharmacological evaluation of these pathways in the context of BD and mood stabilizing treatments may prove fruitful.

## Figures and Tables

**Table 1:** The characteristics of patients with BD and lithium treatment outcomes.

Characteristics BD patients	ConLi^+^Gen	PsyCourse and BipoLife combined
N = 2,558	N = 2,367	N = 191
Good responders to lithium defined as ALDA total score ≥ 7, N (%)	660 (27.9%)	48 (25.1%)
Mean (se) total ALDA score	4.12 (3.15)	4.3 (2.9)
*Country of origin*	N (%)	
Australia	122 (5.2)	
Austria	43 (1.8)	
Canada	353 (14.9)	
Czech Republic	45 (1.9)	
France	210 (8.9)	
German	218 (9.2)	191 (100%)
Italy	255 (10.8)	
Poland	97 (4.1)	
Romania	152 (6.4)	
Spain	74 (3.1)	
Sweden	304 (12.8)	
Switzerland	57 (2.4)	
USA	437 (18.5)	
Age at interview, mean (sd)	47.5 (13.9)	49.1 (13.0)
Sex, Women, N (%)	1369 (57.8)	84 (44.0%)

BD: Bipolar disorder; N: number, sd: standard deviation; se: standard error.

**Table 2:** The association of PGS for lithium variants and treatment response to lithium in patients with BD at different sample splits.

Sample split	N	Categorical outcome OR (95%CI)		Continuous outcome: ALDA total score, OR (95%CI)	
ConLi^+^Gen	2367	unadjusted	adjusted	unadjusted	adjusted^¥^
80%/20%	2083/284	1.31(1.19,1.43)	1.39(1.26,1.54)^¥^	1.15(1.11,1.20)	1.17(1.13,1.22)
Li^+^_PGS_ by decile	^§^R/N				
First (lowest score)	44/236	1 [Reference]	1 [Reference]^¥^	1 [Reference]	1 [Reference]
Second	60/237	1.48(0.96,2.30)	1.59(1.02,2.49)	0.94(0.79,1.12)	0.96(0.81,1.15)
Third	54/237	1.29(0.82,2.02)	1.32(0.84,2.08)	1.07(0.90,1.28)	1.14(0.95,1.35)
Fourth	70/237	1.83(1.19,2.83)	1.87(1.21,2.91)	1.09(0.92,1.31)	1.14(0.96,1.36)
Fifth	59/236	1.45(0.94,2.27)	1.50(0.96,2.35)	1.12(0.93,1.34)	1.17(0.98,1.40)
Sixth	62/237	1.55(1.00,2.40)	1.83(1.17,2.87)	1.22(1.02,1.46)	1.31(1.09,1.55)
Seventh	76/237	2.06(1.35,3.17)	2.27(1.48,3.53)	1.15(0.96,1.38)	1.23(1.04,1.48)
Eighth	68/237	1.76(1.14,2.72)	1.91(1.23,2.99)	1.12(0.93,1.34)	1.17(0.98,1.39)
Nineth	78/237	2.14(1.41,3.29)	2.33(1.51,3.64)	1.45(1.21,1.72)	1.55(1.31,1.86)
Tenth (highest score)	89/236	2.64(1.74,4.05)	3.47(2.22,5.47)	1.52(1.27,1.82)	1.67(1.39,1.99)

The reference decile (1st decile) is the PGS category with the lowest polygenic load for lithium variants. OR (95%CI) for the continuous outcome: ALDA total score is calculated as the exponent of beta coefficient from the linear regression model.

§ R/N: number of lithium responders versus total in that decile;

¥ adjusted for age, sex and 4-genetic principal components, OR: odds ratio.

## Abbreviations

ConLi^+^Gen: the International Consortium on Lithium Genetics
ALDA: Retrospective Criteria of Long-Term Treatment Response in Research Subjects with Bipolar Disorder scale
HRC: Haplotype Reference Consortium (HRC)
SNPs: Single Nucleotide Polymorphisms
MAF: Minor Allele Frequency
GWAS: Genome Wide Association analysis
Li^+^_PGS_: polygenic score for lithium treatment response
LOG: Leave-one-group out procedure
PsyCourse: Pathomechanisms and Signature in the Longitudinal Course of Psychosis study
BipoLife: German research consortium for the study of bipolar disorder
OR: odds ratio
CI: Confidence interval
